# Dynamic Changes in the Intracellular Association of Selected Rab Small GTPases with MHC Class II and DM during Dendritic Cell Maturation

**DOI:** 10.3389/fimmu.2017.00340

**Published:** 2017-03-27

**Authors:** Gibrán Pérez-Montesinos, Orestes López-Ortega, Jessica Piedra-Reyes, Laura C. Bonifaz, José Moreno

**Affiliations:** ^1^Research Unit on Autoimmune Diseases, Research Unit on Immunochemistry, Centro México Nacional Siglo XXI, IMSS, Instituto Mexicano del Seguro Social, Mexico City, Distrito Federal, Mexico; ^2^Centro Dermatológico “Dr. Ladislao de la Pascua”, Secretaría de Salud del Distrito Federal, Mexico City, Distrito Federal, Mexico; ^3^Hospital Juárez de México, Secretaría de Salud, Mexico City, Distrito Federal, Mexico

**Keywords:** major histocompatibility complex class II molecules, Rab GTPases, H2DM, endocytic pathway, dendritic cells

## Abstract

Antigen processing for presentation by major histocompatibility complex class II (MHCII) molecules requires the latter to travel through the endocytic pathway together with its chaperons: the invariant chain (Ii) and DM. Nevertheless, the nature of the compartments where MHCII molecules travel to acquire peptides lacks definition regarding molecules involved in intracellular vesicular trafficking, such as Rab small GTPases. We aimed to define which Rab proteins are present during the intracellular transport of MHCII, DM, and Ii through the endocytic pathway on their route to the cell surface during dendritic cell (DC) maturation. We examined, by means of three-color confocal microscopy, the association of MHCII, DM, and Ii with Rab5, Rab7, Rab9, and Rab11 during the maturation of bone marrow-derived or spleen DC in response to LPS as an inflammatory stimulus. Prior to the stage of immature DC, MHCII migrated from diffuse small cytoplasmic vesicles, predominantly Rab5+Rab7− and Rab5+Rab7+ into a pericentriolar Rab5+Rab7+Rab9+ cluster, with Rab11+ areas. As DC reached the mature phenotype, MHCII left the pericentriolar endocytic compartments toward the cell surface in Rab11+ and Rab9+Rab11+ vesicles. The invariant chain and MHCII transport pathways were not identical. DM and MHCII appeared to arrive to pericentriolar endocytic compartments of immature DC through partially different routes. The association of MHCII molecules with distinct Rab GTPases during DC maturation suggests that after leaving the biosynthetic pathway, MHCII sequentially traffic from typical early endosomes to multivesicular late endosomes to finally arrive at the cell surface in Rab11+ recycling-type endosomes. In immature DCs, DM encounters transiently MHCII in the Rab5+Rab7+Rab9+ compartments, to remain there in mature DC.

## Introduction

The T cell antigen receptor of CD4(+) T cells recognizes peptides bound to major histocompatibility complex class II (MHCII) molecules on the surface of professional antigen-presenting cells (APC) that in primary immune responses are dendritic cells (DCs) (1). DCs initially exist in a steady state (immature), with mostly intracellular MHCII and little peptide diversity. Upon an inflammatory stimulus, in a few hours, DCs become activated and develop into mature DCs with MHCII located at the cell surface and loaded with a great variety of intracellularly acquired peptides as a result of antigen processing in endocytic compartments (2, 3).

Major histocompatibility complex class II α and β chain biosynthesis and translocation into the endoplasmic reticulum is a process guided by the invariant chain (CD74, Ii), which assembles as trimers that permit MHCII assembly (4–7). The resulting MHCII-Ii complexes (up to nonamers) are transported through the Golgi apparatus and the trans-Golgi network (TGN) to the endocytic pathway, where Ii is progressively degraded and removed from MHCII by lysosomal hydrolases (8). Besides guiding MHCII transport throughout the endocytic route, Ii prevents peptide binding to MHCII prior to reaching late endosomes (LE) by means of its class II-associated Ii peptide (CLIP) within residues ~84–105, which covers the MHCII peptide-binding groove (9–12). The final stage of MHCII within the endocytic pathway occurs in a late prelysosomal endocytic compartment, related to multivesicular endosomes, which constitutes the main processing compartment (*MHCII-PC*) (13, 14). Within this organelle, CLIP-loaded MHCII dimers meet DM, a non-classical MHCII-like molecule, which allows CLIP exchange for antigenic peptides onto the MHCII cleft. DM allows an MHCII to bind the best fitting peptide at reach (15), after which peptide-loaded MHCII leave the *MHCII-PC en route* to the cell surface, where they are recognized by CD4(+) T cells.

Intracellular traffic is a tightly regulated process with the participation of several protein families, including soluble *N*-ethyl maleimide sensitive factor (NSF) attachment receptors (SNAREs), adaptors, coating proteins, small GTPases, and among others (16, 17). Of these, Rab and Arf subfamilies of GTPases regulate intracellular traffic, at least in part, by allowing the budding and targeting of transport vesicles and their cargo from donor to specific acceptor compartments (17–19). Rab GTPases and their effectors provide molecular switches that allow the organization of cell-membrane organelles into functional domains that are the sites of arrival and departure of targeted vesicles defined by the presence of particular Rabs and their effectors that coordinate interdomain transport (20–24). Some of the >60 mammalian Rab proteins belong to the endocytic pathway, of which the best characterized are Rab5, found mainly in clathrin-coated endocytic vesicles and early endosomes (EE), playing a role in receptor-mediated endocytosis and EE biogenesis (25–27). Rab7 is in LE and lysosomes (28–30) and, together with Rab5, is found in the transition from EE to LE (31). Rab9 is present in LE and the TGN (32, 33). Finally, Rab11 is characteristic of late recycling endosomes (RE) (21, 34). As yet, most knowledge about Rab GTPases comes from studies on polarized (epithelial) cells. Although a major recent publication has examined in detail the distribution and interactions of all Rab GTPases in DC (35), those studies were achieved in DC lines, with no reference to their functions in different phases of DC differentiation and maturation and their relations to the antigen-processing machinery, such as MHCII and DM arrival to the *MHCII-PC*, as well as MHCII-Ii transport to the *MHCII-PC* and from there to the cell surface. Moreover, additional studies have examined the roles of Rab11 and Rab43 in the phenomenon of cross-presentation for MHCI molecules (36, 37).

Many of the mechanisms of MHCII transport and peptide acquisition after antigen processing have been apparently defined in detail (38). However, several questions remain unanswered, particularly their temporal association with distinct Rab proteins that define particular endocytic compartments. *MHCII-PC* appears closely related to LE, also known as multivesicular bodies (MVBs), but its functional and morphological features resemble the so-called lysosome-related organelles, such as pigment cell melanosomes, endothelial cell Weibel–Palade bodies, platelet α granules, and CTL and natural killer cell lytic granules (39). The study of the association of Rab proteins with the major elements of the MHCII processing machinery during DC maturation is important to further define the nature of *MHCII-PC* among endocytic compartments, the sources and targets of *MHCII-PC* cargo, and the transport of MHCII and their chaperones along the endocytic route to the cell surface. Therefore, we examined the dynamics of MHCII, DM, and Ii temporary association with Rab small GTPases that regulate transport along the endocytic pathway during DC maturation in response to an inflammatory stimulus.

## Materials and Methods

### Mice

B10.BR and C57/BL6j mice used in the experiments were originally purchased from Jackson Laboratories (Bar Harbor, ME, USA). B6-IA^b^-GFP mice have been described (40) and were a generous gift of Drs. Marianne Boes and Hidde Ploegh (Massachusetts Institute of Technology, Boston, MA, USA). Mice were bred and maintained at the Animal Facilities of the Department of Experimental Medicine, Faculty of Medicine, National University of Mexico (UNAM) or at CINVESTAV, Mexico City. The protocol was approved by the National Research and the Ethics Committees of the Coordinación de Investigación, Mexican Institute of Social Security. All experiments were performed *in vitro*, and no manipulation was done to live mice. Four- to eight-week old mice were euthanized by quick immersion in CO_2_. Spleens were removed by postmortem laparotomy, and bone marrow was extracted from femurs and tibias by perfusion with saline through a 25-gauge needle in an insulin syringe.

### Dendritic Cells

For bone marrow-derived DC (BMDC), mouse bone marrow was placed in culture at a density of 5 × 10^5^ cells/mL in RPMI 1640 supplemented with 10% FBS, non-essential amino acids, 2 mM l-glutamine, 100 U/mL penicillin, 100 µg/mL streptomycin, 100 mM HEPES (all from HyClone, Thermo-Fisher Scientific, Logan, UT, USA), and 50 µM 2-mercaptoethanol (Sigma-Aldrich LLC., St. Louis, MO, USA) in the presence of mouse GM-CSF-rich culture supernatants from a GM-CSF-producing cell line (41) (a gift from Dr. Ralph Steinman, Rockefeller University, NY) or recombinant GM-CSF (Merck-Millipore, Darmstadt, Germany) for a 5-day period in bacteriological Petri dishes (Fisherbrand, Thermo-Fisher Scientific, Logan, UT, USA). At day 3 of culture, fresh GM-CSF-supplemented medium (80% of the initial volume) was added to the cultures. At day 5, DCs were highly enriched by means of antimouse CD11c-coated MACS (Miltenyi Biotech, Bergisch Gladbach, Germany) according to manufacturer’s instructions. At this point, DC (~40–60% of total cells) had a predominantly immature phenotype (MHCII^low^/CD86^low^). To induce DC maturation, 100 ng/mL of LPS from *Salmonella typhimurium* (ATCC 14,028 s, courtesy of Dr. Constantino López-Macías, UIMIQ, IMSS) (42) was added to CD11c^+^ cells from 5-day GM-CSF cultures and harvested after varying lengths of time. For some experiments, spleen DCs were isolated by means of antimouse CD11c-coated MACS and similarly cultured in the presence of LPS.

### Antibodies and Reagents

Purified or biotinylated primary antibodies to MHCII (mouse H116.32 and 10.2.16 anti-IA^k^) were from hybridoma culture supernatants (a kind gift of Prof. Gunter Hämmerling, DKFZ, Heidelberg, Germany). Rat NIM-R4 was a courtesy of Dr. Leopoldo Santos-Argumedo, CINVESTAV, Mexico City. In-1 (rat anti-Ii) was a gift of Dr. Norbert Koch. Rat anti-H2-DM, 2C3A, was kindly donated by Dr. Lars Karlsson (Johnson & Johnson Pharmaceutical Research & Development, San Diego, CA, USA) or purchased from Pharmingen (San Jose, CA, USA). Rabbit antisera against Rab5a, Rab7, Rab9, and Rab11a were kind gifts from Dr. Marino Zerial (Max Planck Institute for Cell Biology and Genetics, Dresden, Germany). An additional anti-Rab11a was custom made in sheep immunized with the 20mer peptide RIVSQKQMSDRRENDMSPSN, 174–193 (Invitrogen, Life Technologies, Carlsbad, CA, USA). This peptide, near the c-terminus, is unique to mouse Rab11a and has several sequence differences with any other known Rab proteins, including mouse Rab11b.

Second step reagents were Alexa 660-streptavidin (Molecular Probes, Life Technologies), TRITC- and FITC-antisheep, TRITC- and FITC-antigoat, FITC and TRITC-anti-rat, all from Jackson Immunoresearch Laboratories Inc., PA, USA. Although we mention here the fine specificities of the anti-Rab antisera (Rab5a, Rab11a), there is no evidence that the distinct Rab isoforms (i.e., Rab5a, b, c; Rab7a, B, etc.) play alternative roles in intracellular trafficking, as they only appear to differ in the types of cells expressing them and tissue distribution (43).

### Microscopy

Untreated or LPS-treated DCs were harvested after varying lengths of time and placed in Alcian blue (Fluka, Sigma-Aldrich LLC.)-treated glass slides and were allowed to attach by a 20-min incubation at 4°C, after which they were brought to 37°C for three more minutes. Slides were fixed and permeabilized in BD’s Cytofix/Cytoperm buffer (BD Biosciences, San Jose, CA, USA). Intracellular staining was achieved initially by the addition of a mixture of saturating dilutions of purified or labeled primary antibodies (e.g., MHCII, Rab*n*, DM, Ii) in different combinations, for 30 min, washed 3×, followed by the appropriate mixture of second reagents for each set of primary antibodies for another 30 min, after which they were washed 3× in PBS. Afterward, cells were embedded in VectaShield (Vector Laboratories, Burlingame, CA, USA) and examined immediately or after storage at 4°C, protected from light, until analysis. For most experiments, images were acquired in a Carl Zeiss Axiovert Confocal Microscope equipped with argon 488, HeNe 543, and HeNe 633 nm lasers and 500–530 BP, 565–615 BP, and 650 LP filters with LSM 510 software (Carl Zeiss, Oberkochen, Germany). Image analysis was achieved by means of the LSM 510 software and confirmed using ImageJ (v1.43u, NIH, USA, public domain) and finally processed for quality in Adobe Photoshop CS6 with no manipulations, except for color saturation, sharpness, exposure, contrast, and brightness in entire images. Additional confocal microscope analysis was performed using a Nikon Ti Eclipse confocal microscope equipped with an A1 imaging system, controlled by the proprietary software NIS Elements v.4.50. Imaging was performed using either a 20× (dry, NA 0.8), 60× (oil, NA 1.4), or 100× (oil, NA 1.5) objective lens, as specified in the text. Dyes were excited in a sequential mode using the built-in laser lines: 403 nm (DyLight 405), 488 nm (GFP), 563 nm (Texas Red), and 633 nm (Allophycocyanin). Corresponding fluorescences were read in the following ranges: 425–475 nm (DyLight 405), 500–550 nm (GFP), 570–620 nm (Texas Red), and 663–738 nm (Allophycocyanin).

### Vesicle Distribution Graphs

Early and late stages of MHCII and DM transport and their eventual encounter within the *MHCII-PC* were examined by means of vesicle distribution graphs to represent the distribution of MHCII and DM alone or in combination with Rab5 and/or Rab7 along a line randomly traced through the cell. Pixel intensity information per fluorescence channel was extracted with ImageJ (v1.43u, NIH, USA, public domain). Each graph was generated from a minimum of three DCs at each stage of differentiation per experiment by means of Microsoft^®^ Excel^®^ (v14.3.9, Microsoft Corporation, Redmond, WA, USA). Additional analysis was performed in spleen IA^b^-GFP DCs stimulated with LPS and stained with antibodies against DM, and the rates of colocalization were examined sequentially by ImageJ and Prism softwares.

### Flow Cytometry

Dendritic cell suspensions were stained with the appropriate combination of primary and/or secondary antibodies as described and run in a FACSCalibur flow cytometer (Becton-Dickinson, San Jose, CA, USA). Data analysis was performed by means of the FlowJo software (TreeStar Inc., Ashland, OR, USA).

## Results

### The Distribution of MHCII along the Cytoplasm and Cell Surface Allows the Distinction of Three Stages of DC Differentiation/Maturation

**Figure 1:**
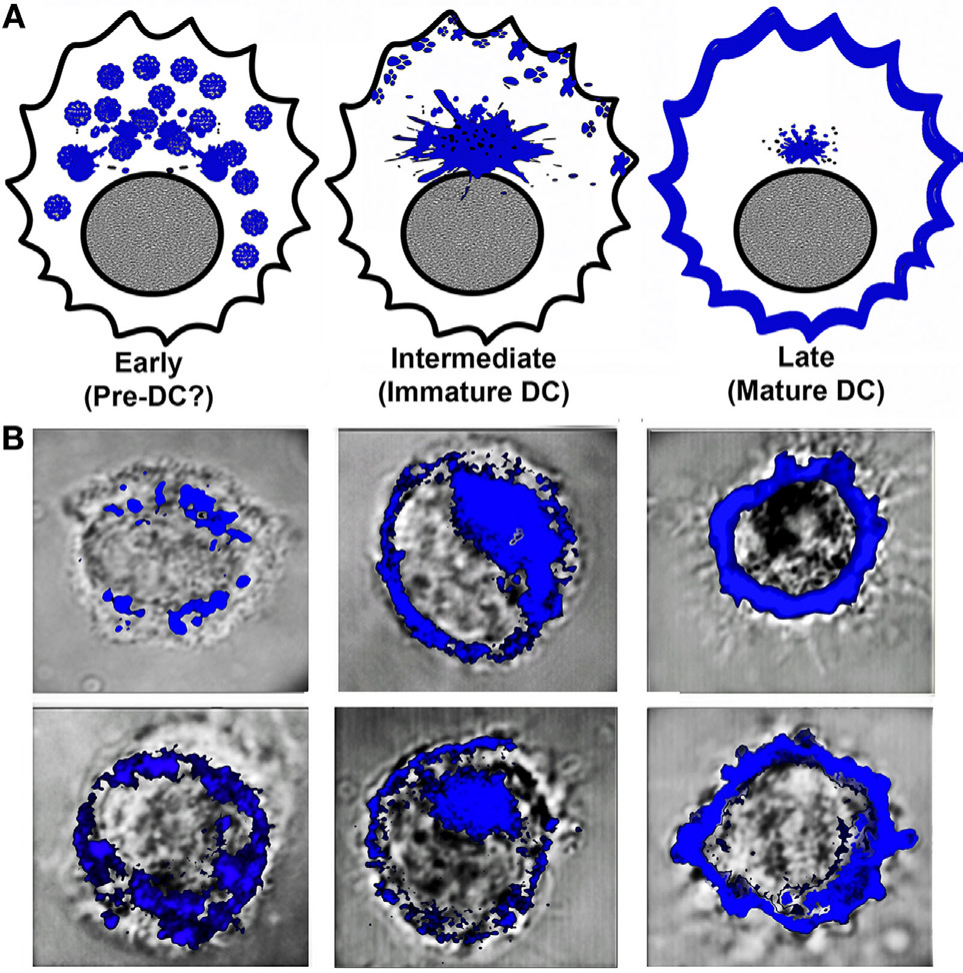
**Stages of dendritic cell (DC) differentiation/maturation according to major histocompatibility complex class II (MHCII) distribution along the cytoplasm and cell surface**. **(A)** Graphical representation of MHCII distribution in DC at three stages of maturation induced by LPS. The cells we call early (left) represent a phase prior to immature DC, are CD11c+, and have no detectable surface MHCII. Intermediate (middle) are typical immature DCs, whereas late (right) are mature DCs. **(B)** Two examples of cells for each stage and their variations, where MHCII in bone marrow-derived DC obtained from B6 mice were visualized in a confocal microscope (100×) in samples stained with antigen-presenting cell-labeled anti-IA^b^.

Given that at any time point of analysis, BMDC cultures contain a mixture of DC phenotypes ranging from pre-DC, immature (intracellular MHCII) to cells fully expressing MHCII on the cell surface (mature DC) (44), to avoid confusion and for better data interpretation we initially defined stages of DC differentiation/maturation by MHCII distribution within the cell. After a 5-day culture in the presence of GM-CSF, BMDCs were cultured in the absence or presence of LPS for varying lengths of time and examined in a confocal microscope for MHCII (Figure 1; Figure S1 in Supplementary Material). Regardless of the length of culture with LPS before analysis, MHCII distribution identifies DCs as follows: the earliest cells (apparently pre-DCs) have small MHCII+ vesicles scattered along the cytoplasm, we refer to them as early DC. Intermediate (immature) DCs contain a large pericentriolar MHCII+ cluster and variable, usually small, amounts of MHCII in the periphery. Finally, mature DCs (referred here to as late) contain large amounts of MHCII in the periphery (mostly at the cell surface) and little or no MHCII in the cytoplasm. In samples not stained for MHCII (i.e., DM), no other marker allowed such distinctions, although in some cells Rab11 had a more or less distinctive pattern of distribution in each of these three stages (Figures 3 and 4). The majority of cells referred here to as early are found in 0–2 h cultures (before or shortly after addition of LPS), intermediate (2–6 h after LPS), and late (usually >6 h after LPS). Although spleen DCs showed similar patterns, the proportions of cells with the described phenotypes were more closely related to the length of culture in the presence of LPS (0–2 and 6 h).

### During the Initial Differentiation of DC, MHCII Merge from Diffuse Cytoplasmic Rab5+Rab7− and Rab5+Rab7+ Small Vesicles into a Large Pericentriolar Rab5+Rab7+ Cluster

**Figure 2:**
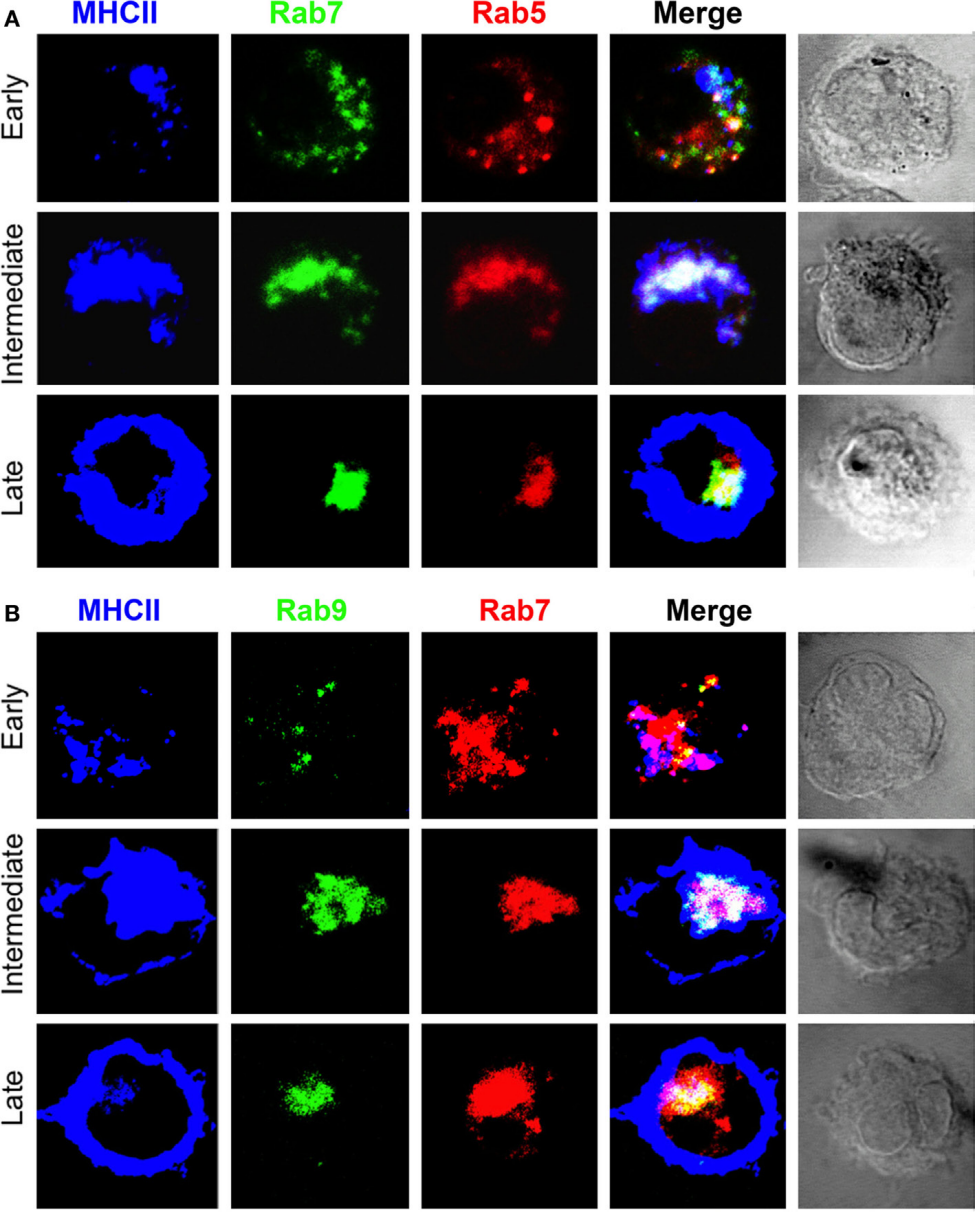
**Progression of major histocompatibility complex class II (MHCII) from cytoplasmic vesicles to pericentriolar compartments but not to the cell surface occurs in concert with Rab5, Rab7, and partial association with Rab9**. Three-color confocal microscope images (100×) of bone marrow-derived dendritic cell from B6 mice untreated (early) or cultured in the presence of LPS (0.1 µg/mL) for at least 8 h and stained for **(A)** MHCII (antigen-presenting cells, APC), Rab5a (TRITC), and Rab7 (Alexa 488) or **(B)** MHCII (IA^b^, APC), Rab7 (TRITC), and Rab9 (Alexa 488). Images are representative of at least three independent experiments.

During DC differentiation, after an inflammatory stimulus, MHCII leave the TGN *en route* to a late endocytic compartment where, with the aid of DM, they bind peptides resulting from partial protein degradation (5, 45). Figure 2A and Figure S2 in Supplementary Material show that in early, unstimulated DC (pre-DC), the majority of MHCII were found in diffuse vesicles scattered along the cytoplasm predominantly colocalizing with Rab5 and Rab7. In intermediate (immature) DCs, MHCII progressively merge into a large pericentriolar cluster, which is heterogeneous, as in its inner part contains Rab5 and Rab7 almost completely overlapping, which is surrounded by an outer MHCII+ rim. At this stage, surface MHCII is absent or low. Finally, in late (mature) DCs, almost all MHCII have left the Rab5+Rab7+ pericentriolar endocytic cluster (PEC) toward the cell periphery, where they are densely expressed, probably both beneath and at the cell surface with no Rab5 and/or Rab7.

### Presence of Rab9 in the MHCII+Rab5+Rab7+ PEC in Immature DC

**Figure 3:**
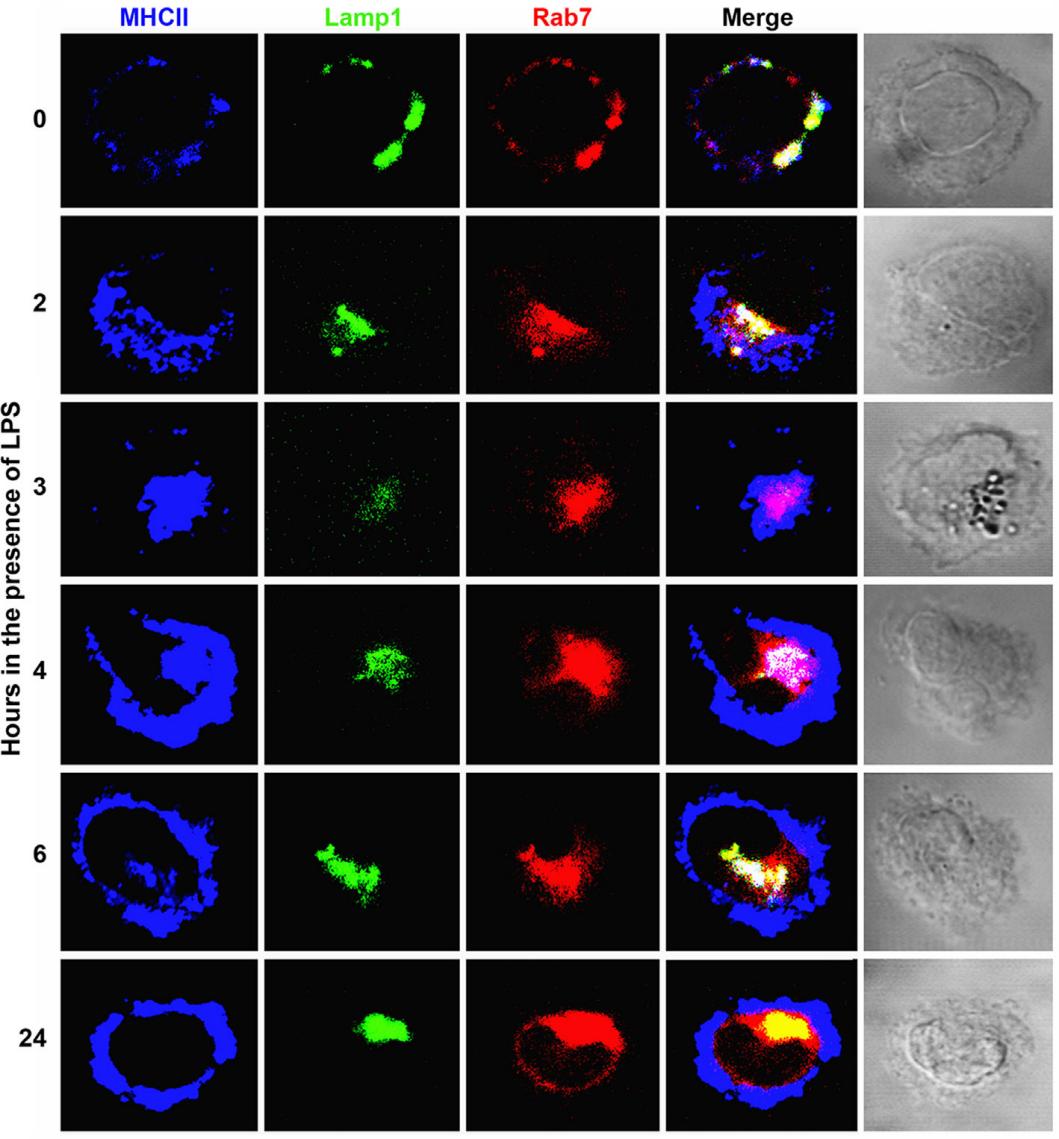
**Near complete colocalization of Lamp1 with Rab7 during dendritic cell (DC) maturation**. Association with major histocompatibility complex class II (MHCII) transport during early to intermediate DC toward pericentriolar compartments (PEC) but not from PEC to the cell surface during intermediate (immature) to late (mature) transition. Three-color confocal microscope images (100×) of bone marrow-derived DC from B10.BR mice, untreated (early) or cultured in the presence of LPS (0.1 µg/mL) for at least 8 h and stained for MHCII (IA^k^, H116.32 plus 10.2.16, followed by donkey anti-mouse-antigen-presenting cells), Rab7 (TRITC), and Lamp1 (Alexa 488). Images are representative of at least three independent experiments.

To better characterize the MHCII+ compartments in early and immature DC, we examined their association with Rab9, a GTPase involved in the recycling of mannose 6-phosphate receptors from LE to the TGN and necessary for MVB biogenesis (33, 46). Figure 2B and Figure S2 in Supplementary Material show that MHCII+ vesicles in early DCs are Rab7+. At the intermediate state, these vesicles have merged with the PEC, which contains Rab9 with a pattern consisting of a central MHCII+Rab7+Rab9+ area surrounded by an MHCII+Rab7+Rab9− rim. In mature (late) DCs, MHCII are present mainly at the cell periphery, whereas Rab7 and Rab9 remain in the PEC with minimal or no MHCII. Of note, Rab7 colocalizes almost entirely with the LE-lysosomal marker Lamp1 (Figure 3). Thus, early transport of MHCII to the endocytic pathway occurs predominantly in small Rab5+Rab7+ cytoplasmic vesicles, a phenotype suggestive of rapidly recycling EE (47). In immature DC, most MHCII are found in a large PEC that contains Rab5, Rab7, and Rab9 heterogeneously distributed, suggesting that such PEC contains discrete, as yet uncharacterized subcompartments. As expected, in mature DC, almost all MHCII but not Rab5, Rab7, and Rab9 are in the cell periphery.

### Late Transport of MHCII to the Cell Surface in Rab11+ Vesicles

**Figure 4:**
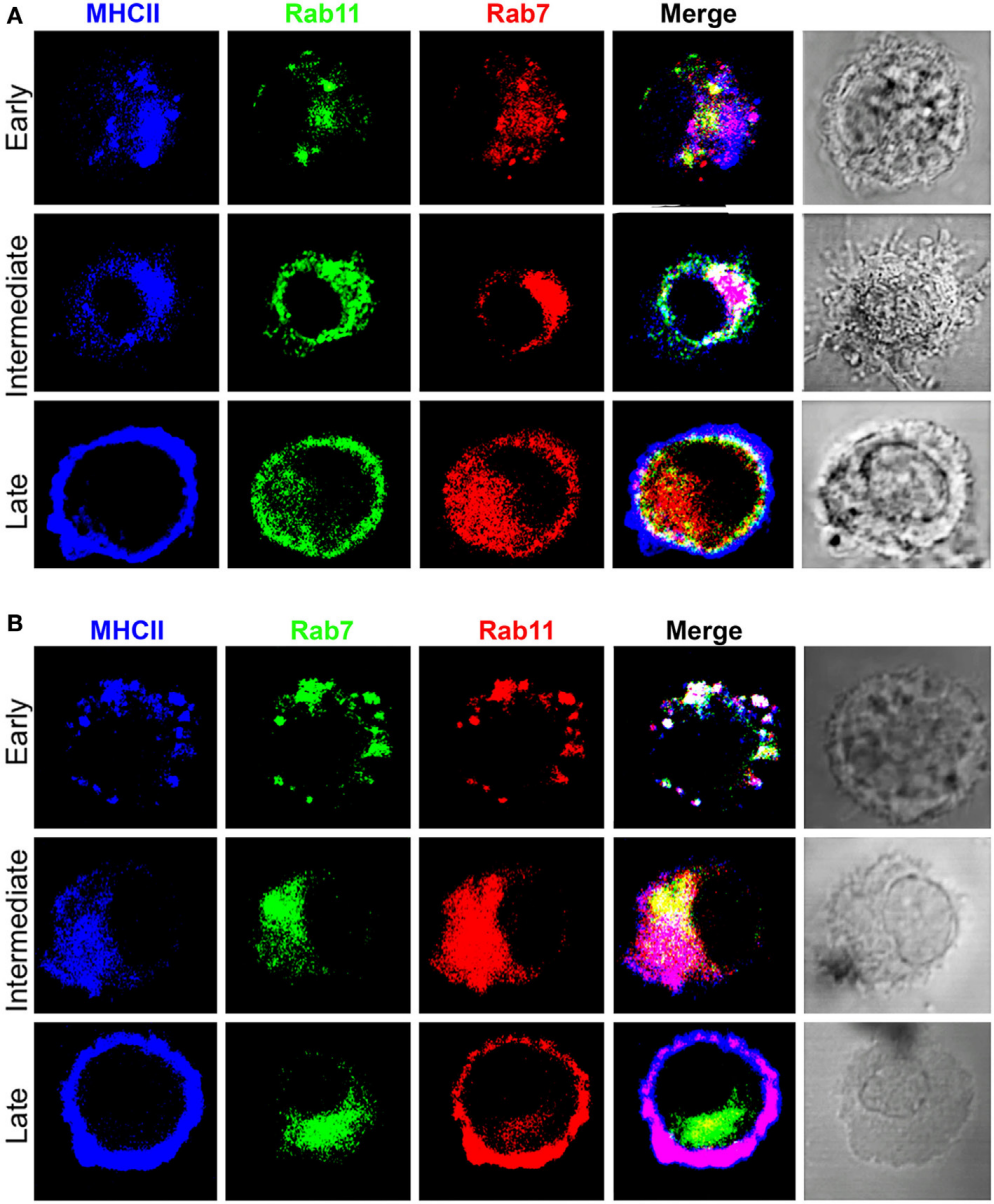
**Transport of major histocompatibility complex class II (MHCII) from the pericentriolar compartment to the cell surface in association with Rab11**. Three-color confocal microscope images (100×) of bone marrow-derived dendritic cell either untreated (early) or cultured in the presence of LPS (0.1 µg/mL) for at least 8 h and stained for **(A)** Expt. 1. B10.BR mice, MHCII [IA^k^, antigen-presenting cells (APC)], Rab11 (TRITC), and Rab7 (Alexa 488), or **(B)** Expt. 2. B6 mice, MHCII (IA^b^, APC), Rab7 (TRITC), and Rab11 (Alexa 488). Images are representative of at least three independent experiments.

Rab11 is a small GTPase involved in vesicle recycling from late and perinuclear endosomes to the plasma membrane (21, 34, 48), making it a useful marker to additionally dissect MHCII transport, particularly at late stages. Figure 4 and Figure S3 in Supplementary Material depict the results of two experiments with different anti-Rab11 antisera showing that before and shortly after the addition of LPS (early DC), the majority of MHCII colocalized again only with Rab7 with some overlap with Rab11. At the intermediate stage, the PEC also contains Rab11 with a heterogeneous distribution, again suggestive of microdomains. In late DCs, the majority of MHCII are in the cell periphery forming an outer MHCII+Rab7−Rab11− rim, apparently in the extracellular side of the plasma membrane, which in some cells surrounds a homogeneous MHCII+Rab11+ internal rim, lying just beneath the cell surface. These results suggest that, during DC maturation, transport of MHCII from the PEC to the cell surface occurs in Rab11+ vesicles, a phenotype typical of late RE.

### Invariant Chain Transport Does Not Faithfully Match MHCII Pathways

**Figure 5:**
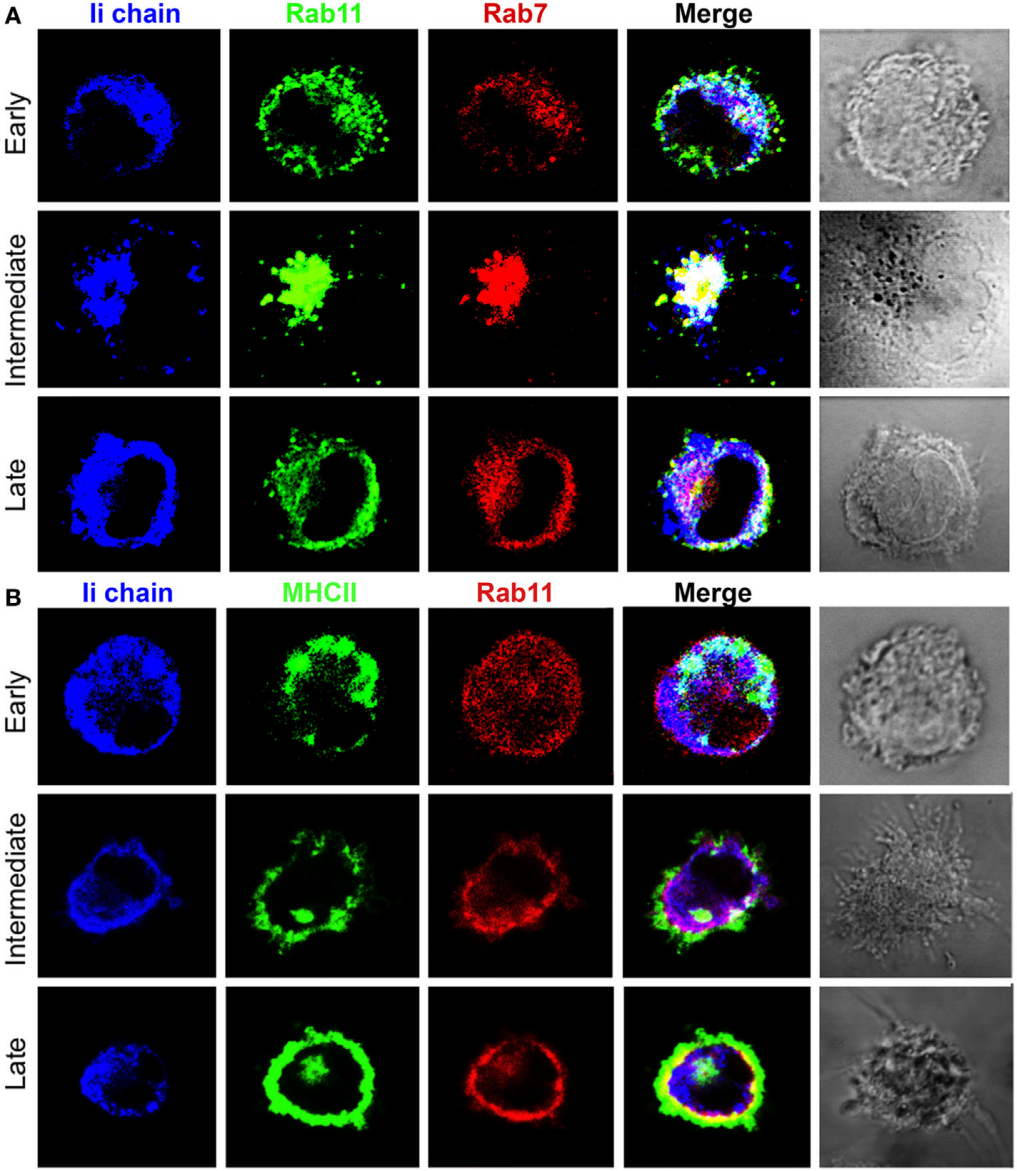
**Partial dissociation of major histocompatibility complex class II (MHCII) and Ii chain transport, distribution, and association with Rab11 at different stages of dendritic cell maturation**. Three-color confocal microscope images (100×) of bone marrow-derived dendritic cell from B6 mouse, either untreated (early) or cultured in the presence of LPS (0.1 µg/mL) for at least 8 h and stained for **(A)** Ii chain antigen-presenting cells (APC), Rab11 (Alexa 488), Rab7 (TRITC) or **(B)** Ii chain APC, MHCII (Alexa 488), Rab11 (TRITC) images are representative of at least three independent experiments.

Next, we examined whether Ii transport had the same patterns as MHCII at each stage of DC maturation. This was important mainly because if the MHCII identified here were of biosynthetic origin, the MHCII+ vesicles in early but not in late DC should be Ii-positive. Figure 5A and Figure S3 in Supplementary Material show that in early DC, as expected, the Ii has a patchy cytoplasmic distribution, which is undistinguishable from MHCII in that it colocalizes with Rab7. At the intermediate state, Ii is mainly in the PEC and associated with Rab7 and Rab11, again similar to MHCII, but with some Ii+ vesicles scattered along the cytoplasm. In contrast, in mature DCs, Ii distribution has a complex pattern, whereas beneath the cell surface it is associated with Rab11, in the remaining cytoplasm, the Ii is diffusely distributed partly colocalizing with Rab11.

When DCs were stained simultaneously for MHCII, Ii, and Rab11 (Figure 5B; Figure S3 in Supplementary Material), in early DCs all MHCII appeared to colocalize with Ii, which, on the other hand, exceeded by far the amount of MHCII with abundant MHCII-free Ii-containing vesicles scattered along the cytoplasm minimally associated with Rab11. In immature DCs, MHCII, Ii, and Rab11 were in the PEC, with abundant free Ii+ vesicles in the cytoplasm. Finally, the most interesting picture was seen in late DC, where MHCII and Ii formed three layers: the most external one (at the cell surface) with only MHCII, the middle layer has both MHCII and Ii overlapping; and finally the most inner (cytoplasmic) layer contains free Ii. This observation suggests that the role of Ii in MHCII transport is more complex than currently thought.

### Diverse Pathways for the Arrival of H2DM and MHCII to the PEC

**Figure 6:**
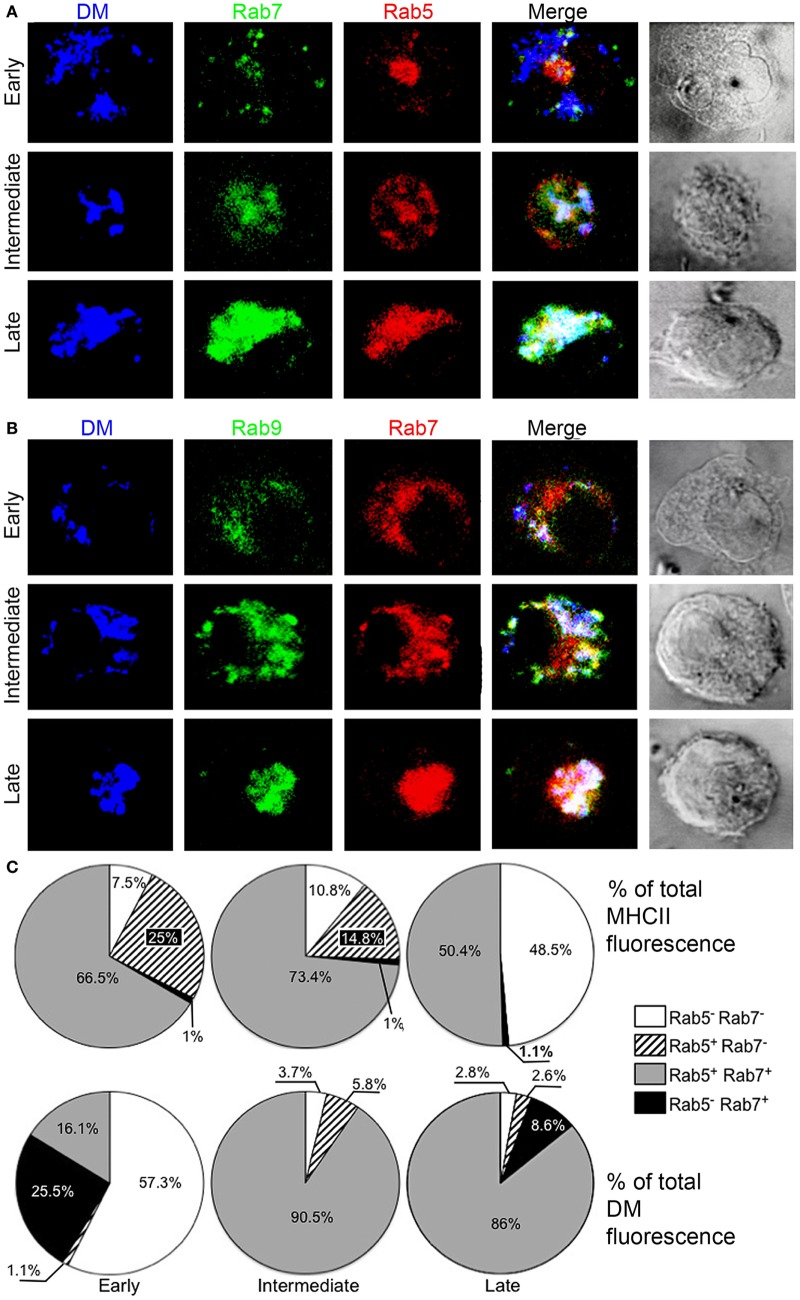
**In early dendritic cells (DCs), DM and major histocompatibility complex class II (MHCII) appear to arrive to PEC through diverse routes**. Three-color confocal microscope images (100×) of bone marrow-derived DC from B6 mice, either untreated (early) or cultured in the presence of LPS (0.1 µg/mL) for at least 8 h and stained for **(A)** DM antigen-presenting cells (APC), Rab7 (TRITC), and Rab5 (Alexa 488) or **(B)** DM APC, Rab9 (Alexa 488), and Rab7 (TRITC). Images are representative of at least three independent experiments. **(C)** DCs from B6 mice processed as above were stained for MHCII (IA^b^, APC, top) or DM (APC, bottom) plus Rab5 and/or Rab7 for three-color confocal microscopy. The distribution of each color was analyzed on individual cell images (≥3 cells) to yield the fluorescence intensity per channel along a straight line, by means of Microsoft^®^ Excel^®^. The percentage far red fluorescence intensity (shown as blue) per pixel (corresponding to either MHCII or DM) along a line traced across the cell was recorded by their coincidence or not with red fluorescence (Rab5) and/or green fluorescence (Rab7). Each graph includes data from at least three DCs at each stage of maturation per experiment. Images are representative of at least three independent experiments.

A major event during MHCII antigen processing is MHCII interaction with the non-peptide binding, non-classical MHCII-like molecule DM that allows peptide exchange onto the MHCII groove and appears to occur mainly in the *MHCII-PC* (15, 49, 50). Hence, we next examined the kinetics of DM transport and association with MHCII during DC maturation. As in previous experiments, BMDCs or spleen DCs were cultured with LPS for different lengths of time and stained for H2-DM plus Rab5, Rab7, and/or Rab9. Figure 6A and Figure S4 in Supplementary Material show that in early DC, similar to MHCII, DM is diffusely located in small cytoplasmic vesicles but, unlike MHCII, many of these are negative for Rab5 and Rab7, with a few DM+ vesicles positive for either GTPases and a minimal fraction colocalizing with Rab9. In intermediate (immature) DCs, most DM is in the typical Rab5+Rab7+PEC that is also Rab9+ (Figure 6B; Figure S4 in Supplementary Material). As opposed to MHCII, in late DCs most DM remained in PEC at 6 h and even at 24 h (the last time point examined). The finding that DM reaches PEC in the stage of immature DC and its association therein with the same Rab proteins as MHCII strongly suggests that such PEC represents the main *MHCII-PC*. Moreover, the differences in the association of MHCII and DM with Rab5 and Rab7 in early DC suggest that DM and MHCII reach the *MHCII-PC* through diverse pathways.

### MHCII and DM Bulk Association with Rab5 and Rab7 during the Initial Stage of DC Maturation Further Suggests Diverse Pathways of Transport

To examine MHCII and DM transport and their eventual encounter within the PEC during DC differentiation/maturation in detail, we created graphs of the distribution of MHCII+ and DM+ vesicles, alone or in combination with Rab5 and/or Rab7, along a line traced across the cell from which we extracted the total pixel intensity per fluorescence channel. Figure 6C shows that in early DC, >90% of MHCII are present in Rab5+Rab7+ or Rab5+Rab7− vesicles (66.6% and 25%, respectively), suggestive of a transition from EE to LE. On the other hand, >50% of DM did not associate with any of these Rab proteins, with 25% of DM in LE (only with Rab7) and 16.1% with both Rab5 and Rab7 in early DCs. In intermediate DCs, the proportion of Rab5−Rab7−-associated DM is similar to MHCII, which, as expected, is even greater in late DCs. In late DCs, most DM (86%) remains in PEC and nearly half MHCII is in Rab5−Rab7− areas.

**Figure 7:**
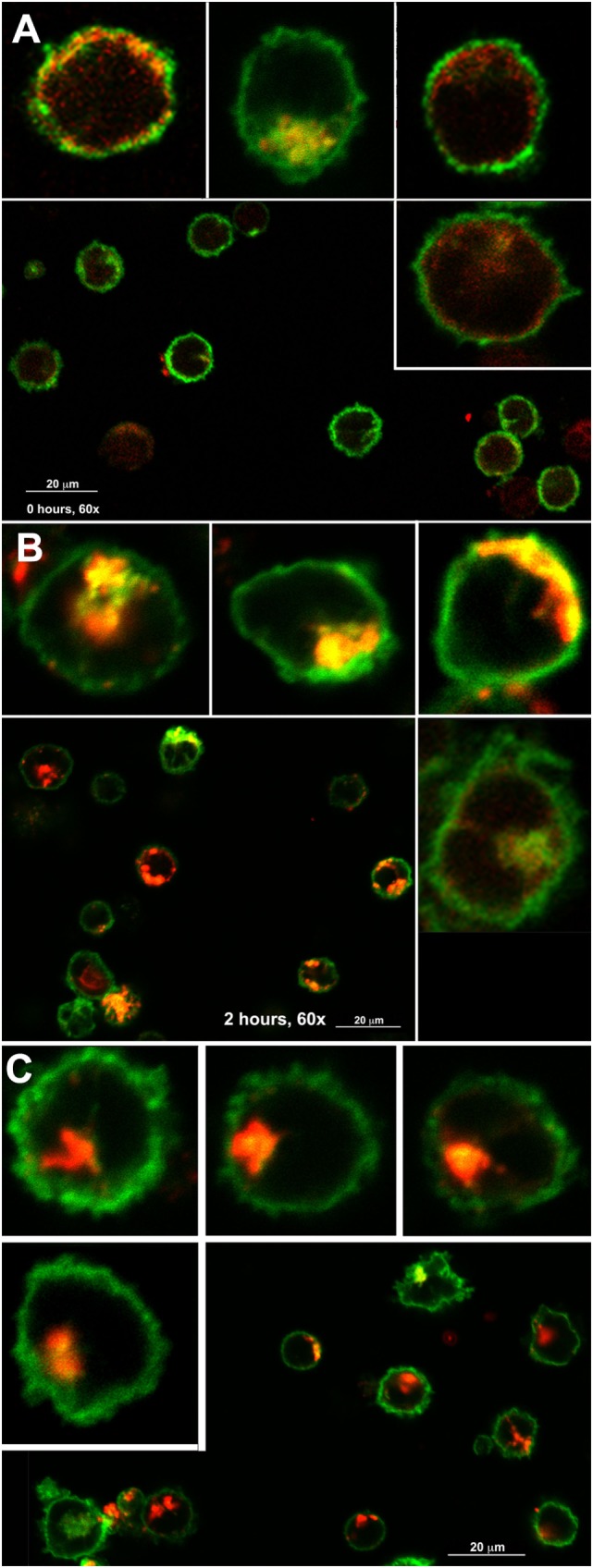
**Major histocompatibility complex class II (MHCII) and DM intracellular transport to the PEC occur through diverse routes**. Confocal microscopy images (60×) of spleen dendritic cells (DCs) from IA^b^-GFP mice before [**(A)**, 0 h], 2 **(B)**, or 6 h **(C)** after the addition of LPS (0.1 µg/mL). Each panel contains a panoramic with varying numbers of DCs, plus four digital enlargements of cells either from the same or from an alternative field, not shown in the panoramic. MHCII (GFP) is shown in green, whereas DM (antigen-presenting cells or DyLight 405) is shown in red. Enlargements are random and do not necessarily represent the actual size of each cell.

**Figure 8:**
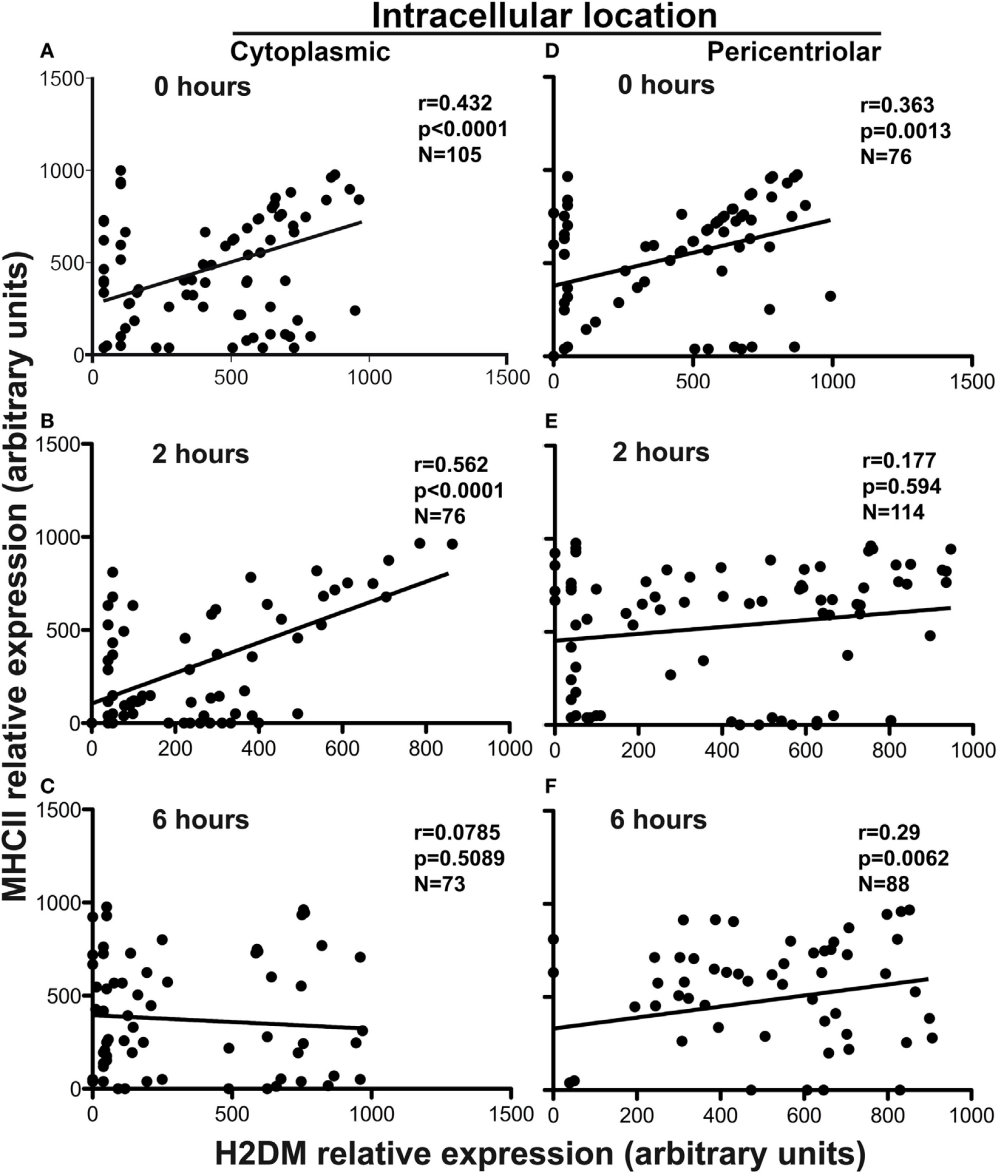
**Dynamics of MHC and DM intracellular transport during dendritic cell (DC) maturation**. Graphics showing the contents of diffuse cytoplasmic vesicles and in the pericentriolar endocytic cluster in DCs before (0 h) **(A,D)** and after culture in the presence of 0.1 µg/mL LPS (2 and 6 h) **(B,C,E,F)**. The data were extracted from confocal microscope analysis of purified B6-IA^b^-GFP spleen DCs stained with anti-DM rat mAb plus antigen-presenting cells (APC)-goat antirat affinity purified antibodies. Relative values of fluorescence intensity for GFP (major histocompatibility complex class II, MHCII) and APC (DM) were quantified from ImageJ software and plotted and examined by means of the Prism 6 software. Statistical data (*r*^2^ and *p*) were obtained by means of the Pearson correlation test. A minimum of 50 cells with at least three GFP+, APC+ or GFP+APC+ dots per cell were examined, and data represent one of three independent experiments.

Finally, we examined the colocalization of MHCII and DM in spleen DC at 0, 2 and 6 h, which, importantly, contain fewer cells of the early phenotype than BMDC. Figure 7 shows confocal microscope images of spleen DCs from IA^b^-GFP mice stained for DM with an APC or DyLight 405-labeled second reagent (shown as red). As seen, early DCs are less abundant than in BMDC, as MHCII (green) is clearly present at the cell surface (Figure 7A). Of the cells shown, only one has larger vesicles that appear to constitute the early phase of the PEC. Nevertheless, in most cells examined (including the four shown), both MHCII and DM were scattered in small vesicles along the cytoplasm, and although there was a significant colocalization of MHCII with DM in cytoplasmic vesicles, there was also a considerable number of MHCII+DM− and MHCII−DM+ vesicles (Figures 8A,B). At 2 h, most cells already contained a pericentriolar cluster (Figures 7B and 8D,E) containing both DM and MHCII with varying degrees of colocalization (the image contains three cells with high level of colocalization and one with an apparently newly formed PEC). Finally, by 6 h, the PEC contains predominantly DM, whereas most MHCII is in the cell periphery. As expected, there was no longer a significant colocalization of MHCII and DM, with only a few MHCII+DM+ vesicles (Figure 8C).

On the other hand, when we examined the degree of colocalization in the pericentriolar area, at 0 h, it contained predominantly MHCII+DM+ dots, together with MHCII+DM− and MHCII−DM+ vesicles, that by 2 h were highly enriched for MHCII and by 6 h were mostly MHCII+DM+, with essentially no MHCII or DM only vesicles (probably due to some MHCII remaining in the PEC). These findings suggest that the pericentriolar area in early and intermediate (immature) DCs is recruiting MHC and DM-containing vesicles, some of which appear to have merged. Moreover, by 2 h, essentially all MHCII vesicles have been recruited to the PEC and at 6 h, the latter although statistically is still present in the PEC (Figure 8F), it has moved predominantly to the cell surface (Figure 7C).

It is possible that in the early spleen DCs, given their more advanced degree of differentiation, which occurred *in vivo*, the incipient PEC contains a higher number of MHCII+DM+ vesicles than in the BM counterparts. Nevertheless, the results suggest that early during DC differentiation/maturation, transport of MHCII and DM appears to occur through diverse vesicular pathways that merge at the intermediate stage (immature DC) prior to reaching the PEC (*MHCII-PC*), at the transition from EE to LE, where DM meets MHCII.

## Discussion

The current studies were undertaken to examine the temporary association of MHCII, DM, and Ii chain with Rab GTPases distinctly expressed in early and late endocytic compartments in LPS-stimulated GM-CSF-induced BM-derived DCs by means of three or four-color confocal microscopy. The major findings of this work are (1) in BMDCs, prior to the stage of immature DC, MHCII merge from diffuse early Rab5+Rab7− and Rab5+Rab7+ endocytic vesicles onto a large late Rab5+Rab7+Rab9+ PEC, typical of immature DC, which also contains Rab11 spots. (2) The arrival of DM to PEC in BMDCs appears to occur through a pathway, which differs slightly from that of MHCII, to engage the endocytic route at a later stage than MHCII in immature DCs. (3) The association of MHCII with Rab11 beneath the cell surface in late (mature) DC suggests a role for recycling perinuclear endosomes in MHCII transport from the PEC to the cell surface. (4) Ii chain transport matches MHCII pathways only partially, as there are abundant MHCII-free Ii chain-containing vesicles, especially in early and late DCs.

Major histocompatibility complex class II biosynthesis and transport is aided by several accessory molecules that act as chaperones for MHCII *en route* to late endocytic processing compartments (6, 7, 51–55). Depending on the maturation state of DCs, *MHCII-PC* appears as a multilamellar (predominant in immature DC) or multivesicular LE (predominant in mature DC) (56). Similar to MVE, vesicular *MHCII-PCs* are compartmentalized by small intraluminal vesicles (ILVs), whereas in multilamellar *MHCII-PCs* ILVs are organized as concentric circles. ILVs carry cytoplasmic fluid immersed in the extracellular fluid of the organelle lumen. Multivesicular *MHCII-PC* resembles MVB, whereas multilamellar *MHCII-PC* appears closer to lysosomes, probably containing MHCII destined for degradation. Regardless of that, there is general acceptance that *MHCII-PC* stands a step prior to lysosomes that are essentially terminal degradative organelles.

It has been suggested that instead of a dedicated *MHCII-PC*, peptide binding to MHCII takes place in a group of related MVBs (57). Analysis of the Rab GTPases present in MHCII+ organelles during DC maturation could contribute to a clearer picture. Very little is known about the relationship of MHCII and antigen processing with Rab proteins; essentially that manipulation of Rab7 (58) or Rab4 (59) alters MHCII-related antigen processing, but, to our knowledge, nothing else. Thus, our present observations add some clarification to the intracellular traffic of MHCII molecules. The PEC found in the cytoplasm of immature DCs appears to be a heterogeneous mixture of LE-related compartments, with the presence of Rab7, Rab9, and Lamp1. The presence of Rab5, which is known to be also present in MVB, suggests that such PEC receives cargo from EE. Moreover, in some cells, the PEC also contains Rab11, which is characteristic of late perinuclear RE (17), suggesting that such spots could be the points of departure for peptide-loaded MHCII molecules to the cell surface. Nevertheless, as parts of the PEC show a complete overlap of Rab5 with Rab7, Rab7 with Rab11, Rab7 with Rab9, and all of them with MHCII or DM, it suggests that at least at some point, all these molecules are simultaneously present at a single site. High-resolution electron microscopy studies have shown that in a late phase of DC maturation, *MHCII-PC*s fuse to form tubular structures (60). It is possible that the distinct vesicles in the PEC exchange their cargo through microtubules, although it would require analysis by ultra-high-resolution microscopy.

The current work also suggests that in early DCs (most likely immature DC precursors), DM and MHCII are transported differently to late endocytic compartments. MHCII is mainly transported in Rab5+Rab7− and Rab5+Rab7+ small vesicles, similar to those that carry exogenous proteins from the extracellular media after phagocytosis or receptor-mediated endocytosis (25, 61–63). Rab5 is characteristic of clathrin-coated endocytic vesicles, EE and can be present in LE, which are Rab7+. Moreover, Rab7 is also present with Rab5 in a subfraction of rapidly maturing EE that are targeted to LE (47). Prior to reaching the endocytic route, newly synthesized MHCII are briefly expressed on the cell surface (64–68). Although we could not detect MHCII on the surface of early DC, it would appear that MHCII are endocytosed in Rab5+ vesicles that fuse with EE. The presence of Ii in these early vesicles is consistent with the idea that such MHCII are of biosynthetic origin. On the other hand, the majority of DM arrived to the PEC in vesicles devoid of all the Rab proteins examined herein, except for a few Rab7+ or Rab9+ vesicles. MHCII and DM are unevenly distributed in the ILVs of MVB (69), such that interaction between them appears to occur only after complete *MHCII-PC* maturation, but how this organization is achieved is not completely clear.

Although the primary role of MHCII is to present peptides from exogenous sources, they often bind peptides of intracellular origin (70). To optimize binding of exogenous peptides, MHCII must be in a peptide-receptive state not prior to late endocytic compartments. This task is achieved in part by Ii, which binds to the MHCII peptide-binding groove through its CLIP region, preventing peptide binding to MHCII from the biosynthetic pathway until EE (9). Ii is degraded stepwise by lysosomal proteases until the *MHCII-PC*, wherein only the CLIP remains bound. Once in *MHCII-PC*, DM interacts with MHCII-CLIP complexes, allowing the release of CLIP. The finding that at least some DM and MHCII are delivered separately to late endocytic compartments could explain their spatial separation within the MVE.

Two final and probably relevant findings of the current studies are (1) the association of Rab11 and MHCII in the periphery of mature DC, just beneath the cell surface and (2) the unexpected distribution of MHCII-free Ii in late DC. As for the former, Rab11 is typical of a subset of recycling pericentriolar endosomes, known to play important roles in exocytosis of a number of molecules in different cell types exocytosis (48, 71, 72), and even in late MHCI transport during cross-presentation (36). Thus, it is not surprising to have found this GTPase in association with MHCII in late stages of DC maturation, suggesting a role for Rab11+ vesicles in MHCII transport from *MHCII-PC* to the cell surface. This differs from the current notion that the limiting membrane of MVB or *MHCII-PC* fuses in its entirety with the plasma membrane to deliver peptide-loaded MHCII (3, 38), whereas ILV-associated MHCII is either targeted to lysosomes for degradation or released as exosomes when MVB fuses with the plasma membrane (73). All late DCs examined here had MHCII associated only with Rab11 in the cell periphery, whereas Rab5, Rab7, and Rab9 remained in the PEC that persisted after DC maturation, suggesting that the PEC does not fuse to the plasma membrane; instead, that, prior to expression at the cell surface, MHCII are contained in Rab11+ vesicles, most likely RE, beneath the plasma membrane. It has been shown that MHCII are transferred to the plasma membrane through tubular structures that appear to be elongations of the *MHCII-PC* or MVB, which associate with MHCII-enriched vesicles in its most external regions (74), Rab11 could be part of such structures. The PEC described here for immature DC contained Rab11+ patches, that in mature DC were not seen, but Rab11 was, instead, in the periphery. This is consistent with the recently described analysis of Rab11a distribution in DC lines (35). If the images we show here represent tubular elongations of *MHCII-PC* or MVB, Rab5, Rab7, and Rab9 are absent in them.

Finally, the association of Rab11 with Ii in the periphery of late DC is intriguing, and it is unexpected too to find Ii-associated MHCII near the cell surface in mature DCs. As the In-1 mAb used here is directed at the Ii cytoplasmic tail, we do not know whether such Ii is a complete molecule or a partially degraded intermediate fragment. The Ii is known to perform some non-MHCII-related functions, including association with MHCI (75, 76), CD1 (77), and CD44 (78), or as cytokine receptor for the macrophage inhibitory factor (79). Thus, it is possible that MHCII-free Ii chain is performing non-MHCII-related or any other, as yet, unknown chaperone function.

## Ethics Statement

This study was carried out in accordance with the recommendations of the Comité de Ética e Investigación, Coordinación de Investigación en Salud, IMSS. All *in vitro* studies, with no procedures performed to live animals. Mice were euthanized by quick immersion in CO_2_.

## Authors Note

This work was accomplished to fulfill the requirements of GP-M to obtain his Ph.D. degree at the Doctorate Program on Biomedical Sciences of the Universidad Nacional Autónoma de México.

## Author Contributions

GP-M performed the experiments, helped in the writing of the manuscript, and analyzed the images. OL-O and JP-R performed confocal microscopy experiments and did the statistical analysis contributing with all data for Figures 7 and 8. LB helped in the writing of the manuscript, analyzed the images, discussed, and helped to interpret the data. JM conceived the study, wrote the manuscript, supervised the experimental procedures, and analyzed and interpreted the images.

## Conflict of Interest Statement

The authors declare that the research was conducted in the absence of any commercial or financial relationships that could be construed as a potential conflict of interest. The reviewer BM and handling editor declared their shared affiliation, and the handling editor states that the process nevertheless met the standards of a fair and objective review.

## Abbreviations

MHCII-PC, MHCII-related processing compartment; NSF, soluble *N*-ethyl maleimide sensitive factor; LRO, lysosome-related organelles; PEC, pericentriolar compartments and pericentriolar endocytic cluster (interchangeable).
