# Supplementary material for: Dynamic Changes in the Intracellular Association of Selected Rab Small GTPases with MHC Class II and DM during Dendritic Cell Maturation
Source: Front Immunol. 2017 Mar 27;8:340. doi: 10.3389/fimmu.2017.00340 (PMC5367080; doi:10.3389/fimmu.2017.00340)
Supplement: Supplementary file 1 [file Presentation_1.PDF]

Supplementary figures and legends

Figure 1s. Panoramic view MHCII distribution along the cytoplasm and cell surface at three different DC differentiation/maturation stages. Examples of dendritic cell cultures stained with anti-IA^{b}-APC after a 0 (left), 2 (middle) and >6 hours (right) culture in the presence of LPS (0.1 μg/mL), showing the dominant MHCII distribution phenotypes: early, intermediate and late, respectively. MHCII were visualized (40x) in a confocal microscope.

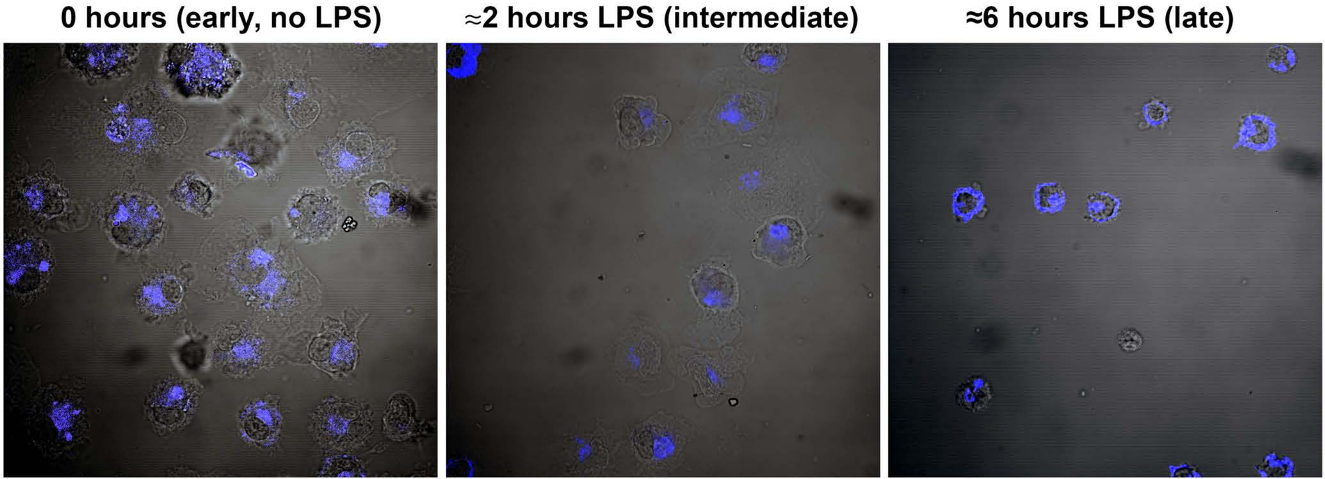
0 hours (early, no LPS)
≈2 hours LPS (intermediate)
≈6 hours LPS (late)

Figure 2s. Progression of MHCII+ to pericentriolar compartments but not to the cell surface in Rab5+Rab7+Rab9+/- cytoplasmic vesicles. Three-color confocal microscope images (100x) of BMDC untreated (early) or cultured in the presence of LPS (0.1 μg/mL) for at least 8 hours and stained for: **A)** MHCII (APC), Rab5a (TRITC) and Rab7 (Alexa 488), or **B)** MHCII (APC), Rab7 (TRITC) and Rab9 (Alexa 488). Images are representative of at least three independent experiments.

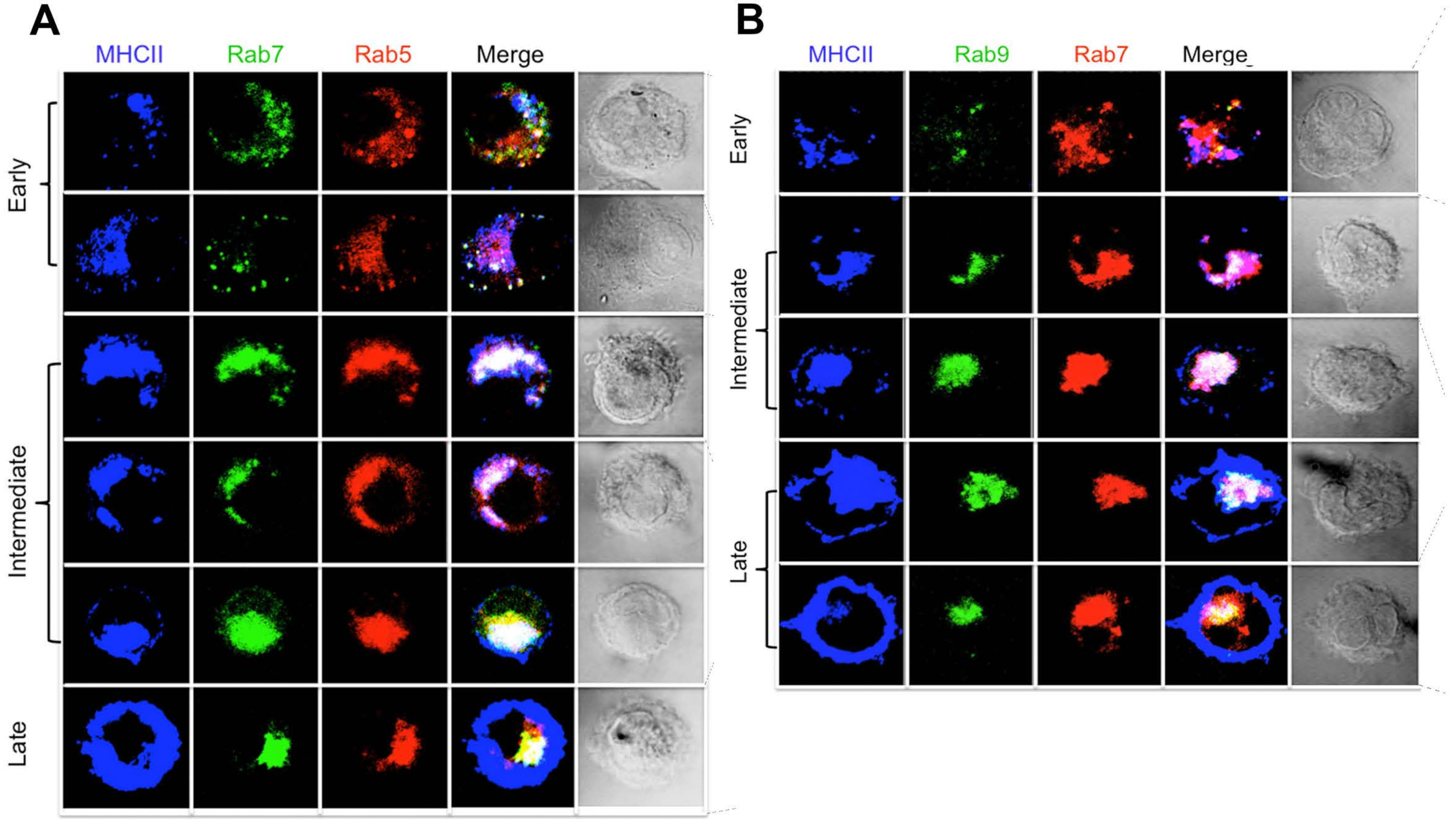
A
MHCII
Rab7
Rab5
Merge
Early
Intermediate
Late
B
MHCII
Rab9
Rab7
Merge
Early
Intermediate
Late

Figure 3s. Transport of MHCII from pericentriolar compartments to the cell surface in Rab11+ vesicles and partial dissociation of MHCII and Ii chain transport, distribution, and association with Rab11 in different stages of DC maturation.. Three-color confocal microscope images (100x) of BMDC either untreated (early) or cultured in the presence of LPS (0.1 μg/mL) for at least 8 hours and stained for: **A)** Expt. 1. MHCII (APC), Rab11 (TRITC) and Rab7 (Alexa 488), or **B)** Expt. 2. MHCII (APC), Rab7 (TRITC) and Rab11 (Alexa 488). **C)** Ii chain (APC), Rab11 (Alexa 488), Rab7 (TRITC), and **D)** Ii chain (APC), MHCII (Alexa 488), Rab11 (TRITC). Images are representative of at least three independent experiments.

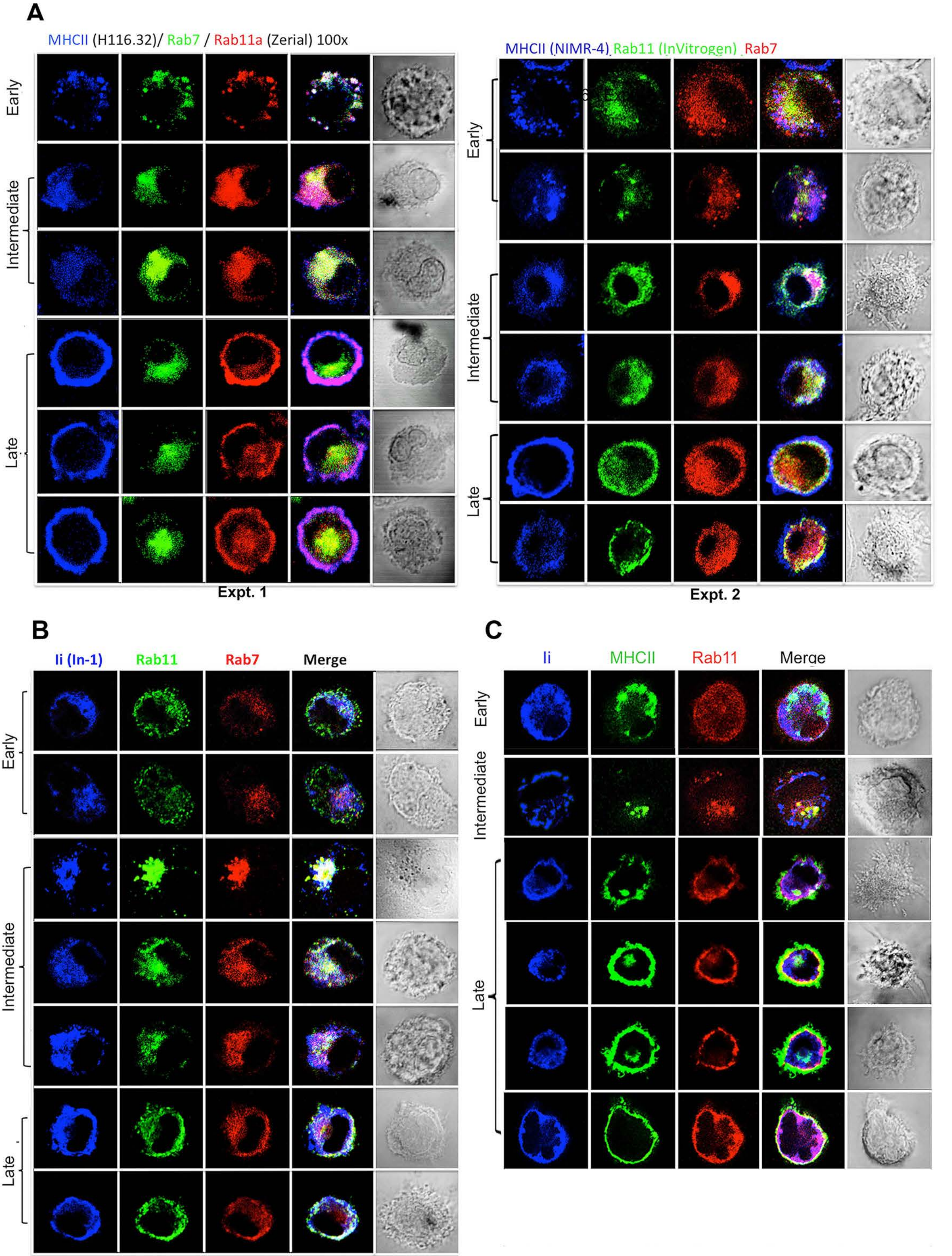
A
MHCII (H116.32)/ Rab7 / Rab11a (Zerial) 100x
Early
Intermediate
Late
Expt. 1
MHCII (NIMR-4) Rab11 (InVitrogen) Rab7
Early
Intermediate
Late
Expt. 2
B
Ii (In-1)
Rab11
Rab7
Merge
Early
Intermediate
Late
C
Ii
MHCII
Rab11
Merge
Early
Intermediate
Late

Figure 4s. **In early DCs, DM and MHCII arrive to PEC through independent routes.** Three-color confocal microscope images (100x) of BMDC either untreated (early) or cultured in the presence of LPS (0.1 μg/mL) for at least 8 hours and stained for: **A)** DM (APC), Rab7 (TRITC) and Rab5 (Alexa 488) or **B)** DM (APC), Rab9 (Alexa 488) and Rab7 (TRITC). Images are representative of at least three independent experiments.

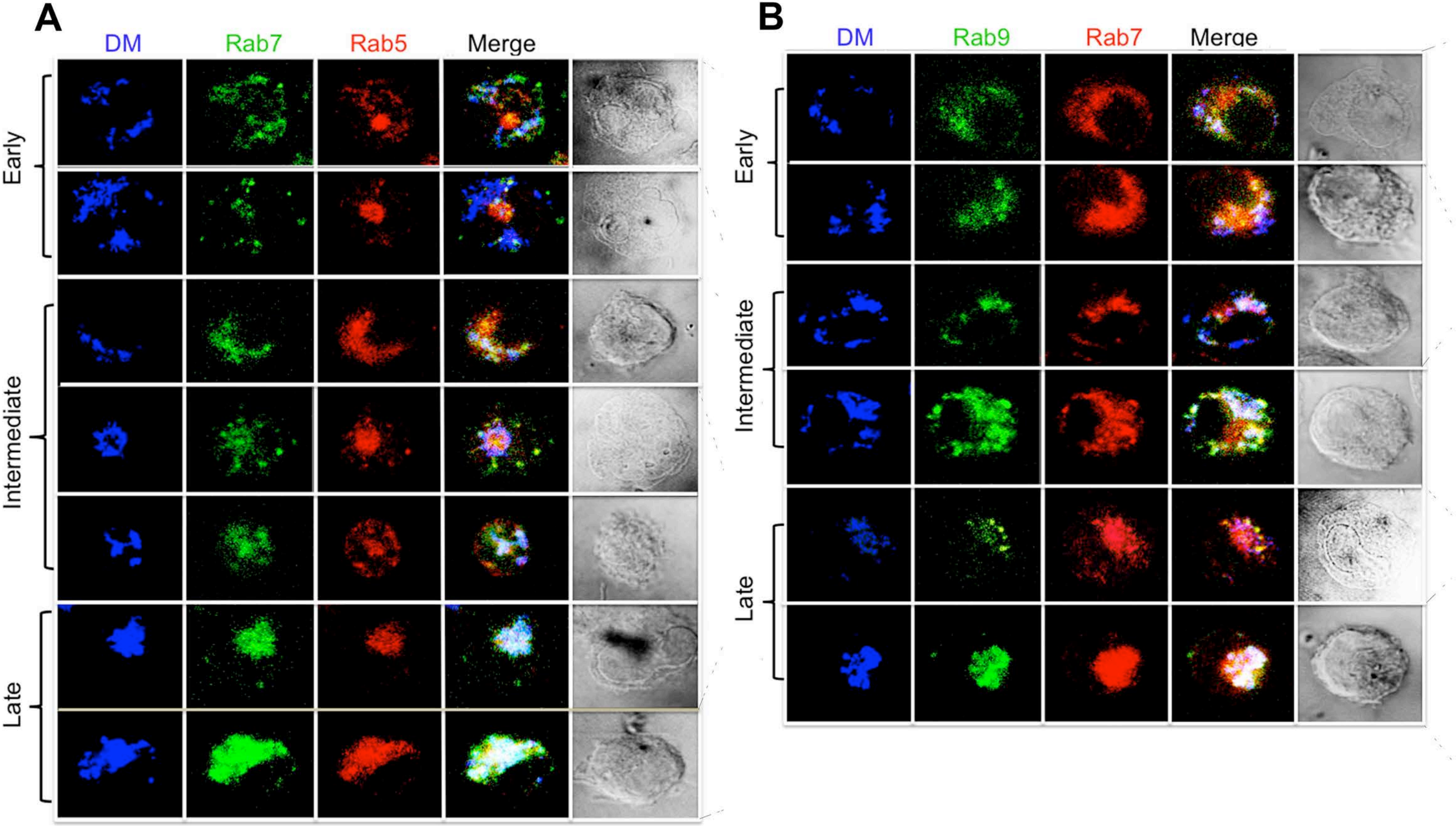

A
DM
Rab7
Rab5
Merge
Early
Intermediate
Late
B
DM
Rab9
Rab7
Merge
Early
Intermediate
Late
